# Risk of ischemic stroke and utility of CHA_2_DS_2_‐VASc score in women and men with atrial fibrillation

**DOI:** 10.1002/clc.23257

**Published:** 2019-09-06

**Authors:** Maria Tomasdottir, Leif Friberg, Ziad Hijazi, Johan Lindbäck, Jonas Oldgren

**Affiliations:** ^1^ Department of Medical Sciences Uppsala University Uppsala Sweden; ^2^ Department of Clinical Sciences at Danderyd Hospital Karolinska Institute Stockholm Sweden; ^3^ Uppsala Clinical Research Center Uppsala University Uppsala Sweden

**Keywords:** atrial fibrillation, CHA_2_DS_2_‐VASc risk score, ischemic stroke

## Abstract

**Background:**

The magnitude of increased risk of stroke in women with atrial fibrillation (AF) remains uncertain.

**Hypothesis:**

We investigated the risk of ischemic stroke and death in women and men with AF, and the risk associated with individual non‐sex CHA_2_DS_2_‐VASc risk factors.

**Methods:**

Retrospective cohort study of 231 077 (48.1% women) nonselected patients with AF not receiving oral anticoagulation from 2006 to 2014. Data from cross‐linked national Swedish registers. The outcome was the first occurrence of ischemic stroke or death. Median age was 82 and 75 years in women and men, respectively. Mean follow‐up was 2.5 years.

**Results:**

Hazard ratios, adjusted for non‐sex CHA_2_DS_2_‐VASc risk factors, for women vs men were 1.53, 95% CI: 1.49‐1.58 for ischemic stroke and 1.24, 95% CI: 1.22‐1.26 for death, respectively. When divided into age groups the differences in ischemic stroke rates between women and men were attenuated. In patients with only one non‐sex CHA_2_DS_2_‐VASc risk factor allotted 1 point, ischemic stroke rates per 100 person‐years were 1.22 in women (n = 9838) and 1.02 in men (n = 15 609), respectively, *P* < .006. In both women and men, age of 65 to 74 years was associated with higher ischemic stroke risk compared to other non‐sex CHA_2_DS_2_‐VASc risk factors allotted 1 point.

**Conclusions:**

The risk of ischemic stroke was 1.5‐fold higher in women compared to men but this association appears to be the result of confounding by age. In the low risk end, the CHA_2_DS_2_‐VASc risk score underestimates the ischemic stroke risk conferred by age 65 to 74 years, while it overestimates the risk conferred by female sex.

## 1 INTRODUCTION

To assess the risk of stroke in patients with atrial fibrillation (AF), international guidelines recommend the use of the CHA_2_DS_2_‐VASc risk scoring system to identify those who may benefit from oral anticoagulation (OAC) treatment.^1^, ^2^ Studies have shown conflicting results regarding the magnitude of increased stroke risk associated with each of the variables in the CHA_2_DS_2_‐VASc score, especially regarding female sex.^3^, ^4^, ^5^, ^6^, ^7^, ^8^, ^9^, ^10^, ^11^, ^12^ Further research on the risk of stroke association of female sex and stroke risk is therefore needed and was encouraged by the European AF guidelines.^1^

We aimed to (a) investigate the risk of ischemic stroke and death in women and men with AF and (b) assess the risk for ischemic stroke associated with each component of the CHA_2_DS_2_‐VASc score separately in women and men with one additional non‐sex CHA_2_DS_2_‐VASc risk factor.

## 2 METHODS

### 2.1 Data

This was a retrospective nationwide Swedish cohort study. Data were collected from The National Patient Register cross‐linked with The Dispensed Drug Register and The Cause of Death Register including all permanent residents in Sweden. For the identification of patients and record linkage, we used individual civic registration numbers. The Patient Register includes detailed information about all hospitalizations and visits to hospital outpatient clinics, with diagnoses coded according to the International Classification of Diseases (ICD). The Dispensed Drug Register stores details about every dispensed prescription in Sweden since 1 July 2005. All pharmacies in the country are required to participate by law, and information is transferred electronically whenever a drug is dispensed. The Dispensed Drug Register does not contain information about prescriptions that have not been dispensed, drugs used during hospital stay or over‐the‐counter drugs. The Cause of Death Register includes diagnostic information about causes and contributory causes of death for those living in Sweden at time of death.

### 2.2 Study population

The cohort included all patients with an AF diagnosis registered between 2 December 2005 and 31 December 2014. For each patient, the first day of follow‐up was set as 30 days after the first occurrence of an AF diagnosis during the inclusion period. This clearance period is used to prevent an ischemic stroke event that happens before, or at the time of, AF diagnosis being counted as an outcome event, as has been described in previous publications.^13^, ^14^ Follow‐up ended on 31 December 2014. A flowchart of the included patients is shown in Figure S1. Patients younger than 18 years and those with mitral stenosis or a prosthetic heart valve were excluded. The ICD codes used for exclusion are listed in Table S1. Patients who had filled a prescription for an OAC within 6 months prior to the start date of the study were excluded and patients who filled a prescription for an OAC during the follow‐up period were censored at that time. The medication codes used for defining OAC medications are listed in Table S1.

### 2.3 Outcomes and estimation of CHA_2_DS_2_‐VASc risk factors

The outcome was the first of either ischemic stroke or death. CHA_2_DS_2_‐VASc risk factors were identified by ICD codes in the Patient Register, with addition of diabetes medication codes in the Dispensed Drug Register. CHA_2_DS_2_‐VASc risk factors at baseline were estimated only from diagnoses given before index date.

### 2.4 Statistical methods

The analysis was performed in several steps. First, unadjusted incidence rates per 100 person‐years with 95% confidence interval (CI) were estimated assuming the number of events within specific subgroups followed a Poisson distribution with constant event rate during the follow‐up period. Patients were censored at the time of a filled prescription for an OAC, at the end of follow‐up and, in the analysis of the endpoint of ischemic stroke, at death. Patients were not censored if and when they developed new comorbidities under the study period. Within each CHA_2_DS_2_‐VASc category, a statistical comparison between men and women was done by fitting a Cox regression model, thus relaxing the assumption of constant absolute event rates including gender as the only variable. Second, to account for competing risk of death, cumulative incidence rates were calculated by estimating cumulative subdistributions for the separate endpoints ischemic stroke and death, respectively. Third, to describe the event rate as a continuous function of age for women and men, a multistate Poisson model was fitted with death and ischemic stroke as possible endpoint states, thus accounting for competing risks, and including gender, age, and the interaction between gender and age, where age was represented as a natural spline with knots at 40, 50, 60, 70, 80, and 90 years, together with the remaining CHA_2_DS_2_‐VASc risk factors: heart failure, hypertension, diabetes, stroke, transient ischemic attack, or systemic embolism and vascular disease. The reason for fitting a Poisson model instead of a Cox model, which might seem more natural for time‐to‐event data, was that the Poisson approach allows for a direct parametric modeling of the baseline rate and also for analyzing multiple time scales; in our case time from study entry and age. The age time scale was split in 5‐year intervals in which the rates were assumed constant. All analyses were done with R,^15^ version 3.3.2 using the Epi package.^16^

## 3 RESULTS

**Figure 1:**
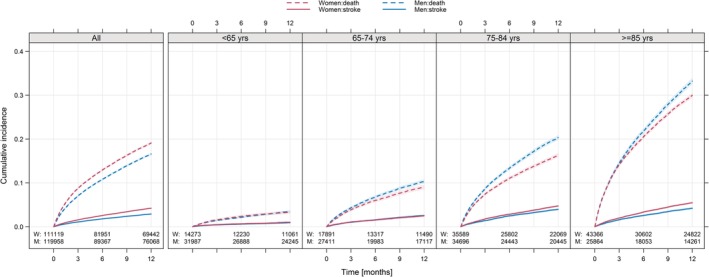
Cumulative incidence of ischemic stroke and death. Cumulative incidence of ischemic stroke (solid lines) and death (broken lines) in women (red lines) compared to men (blue lines). Shaded area represents 95% confidence intervals. The total group of the 231 077 included patients is shown to the far left and then divided into four different age groups to the right. Follow‐up limited to 12 months. M, number of men; W, number of women

A total of 231 077 patients were identified of which 111 119 (48.1%) were women. The median age was 79 years; 75 years for men and 82 years for women, respectively. The mean follow‐up was 2.5 years. The baseline characteristics by gender for all included patients are shown in Table S2. The ischemic stroke incidence rate per 100 person‐years was 2.38 (95% CI: 2.32‐2.43) for men and 3.69 (CI: 3.62‐3.76) for women with a total number of events of 7415 in men and 10 125 in women, respectively. The incidence rate of death was 13.80 (CI: 13.67‐13.93) for men and 17.79 (CI: 17.63‐17.94) for women with total number of events of 44 375 in men and 50 971 in women, respectively. Despite an overall higher risk of ischemic stroke and death in women compared to men (Figure 1, left panel), when divided into age groups the differences in ischemic stroke rates between women and men were attenuated in the younger age groups, while men had higher rates of death in the older age groups (Figure 1, right panel).

Also, cause‐specific hazard ratios (HRs) for the two competing risk outcomes (ischemic stroke and death) for women compared to men are shown in Table 1.

**Table 1:** Cause‐specific hazard ratios (HRs; with 95% confidence intervals) for the two competing risks outcomes ischemic stroke and death, and for the composite endpoint of ischemic stroke or death for women compared to men

Age (y)	Sex	N	Stroke	HR	Death	HR	Stroke or death	HR
<65	Female	14 273	400	1.13 [1.00, 1.27]	995	0.89 [0.82, 0.95]	3242	0.95 [0.89, 1.01]
	Male	31 987	780		2462		1395	
65‐74	Female	17 891	1051	1.03 [0.95, 1.12]	2972	0.79 [0.76, 0.83]	7010	0.84 [0.81, 0.88]
	Male	27 411	1492		5518		4023	
75‐84	Female	35 589	3778	1.15 [1.09, 1.20]	13 262	0.80 [0.78, 0.82]	17 874	0.85 [0.84, 0.87]
	Male	34 696	2959		14 915		17 040	
≥85	Female	43 366	4896	1.25 [1.19, 1.32]	27 167	0.87 [0.85, 0.88]	19 575	0.91 [0.89, 0.93]
	Male	25 864	2184		17 391		32 063	
All	Female	111 119	10 125	1.53 [1.49, 1.58]	44 396	1.24 [1.22, 1.26]	47 701	1.28 [1.27, 1.30]
	Male	119 958	7415		40 286		54 521	

*Note*: All hazard ratios are adjusted for the CHA_2_DS_2_‐VASc risk factors: heart failure, hypertension, diabetes, previous stroke/TIA/systemic embolism and vascular disease. The model for all ages was also adjusted for age groups. N, number of patients.

Overall, women had a higher risk of ischemic stroke as compared with men. The relative risk increase in women was less pronounced of those aged 65 to 74 years (HR: 1.03, CI: 0.96‐1.12 for women vs men) as compared those aged 85 years or older (HR: 1.25, CI: 1.19‐1.32). Overall, women had a higher risk of death (HR: 1.24, CI: 1.22‐1.26), although this pattern did not remain when evaluating different age groups, where women actually had a lower risk of death in all age groups (Table 1). These findings were also similar for the composite endpoint of stroke or death, in which women had an overall higher risk than men (HR: 1.28, CI: 1.27‐1.30), but when divided into different age groups women had lower risk in all age groups (Table 1).

**Figure 2:**
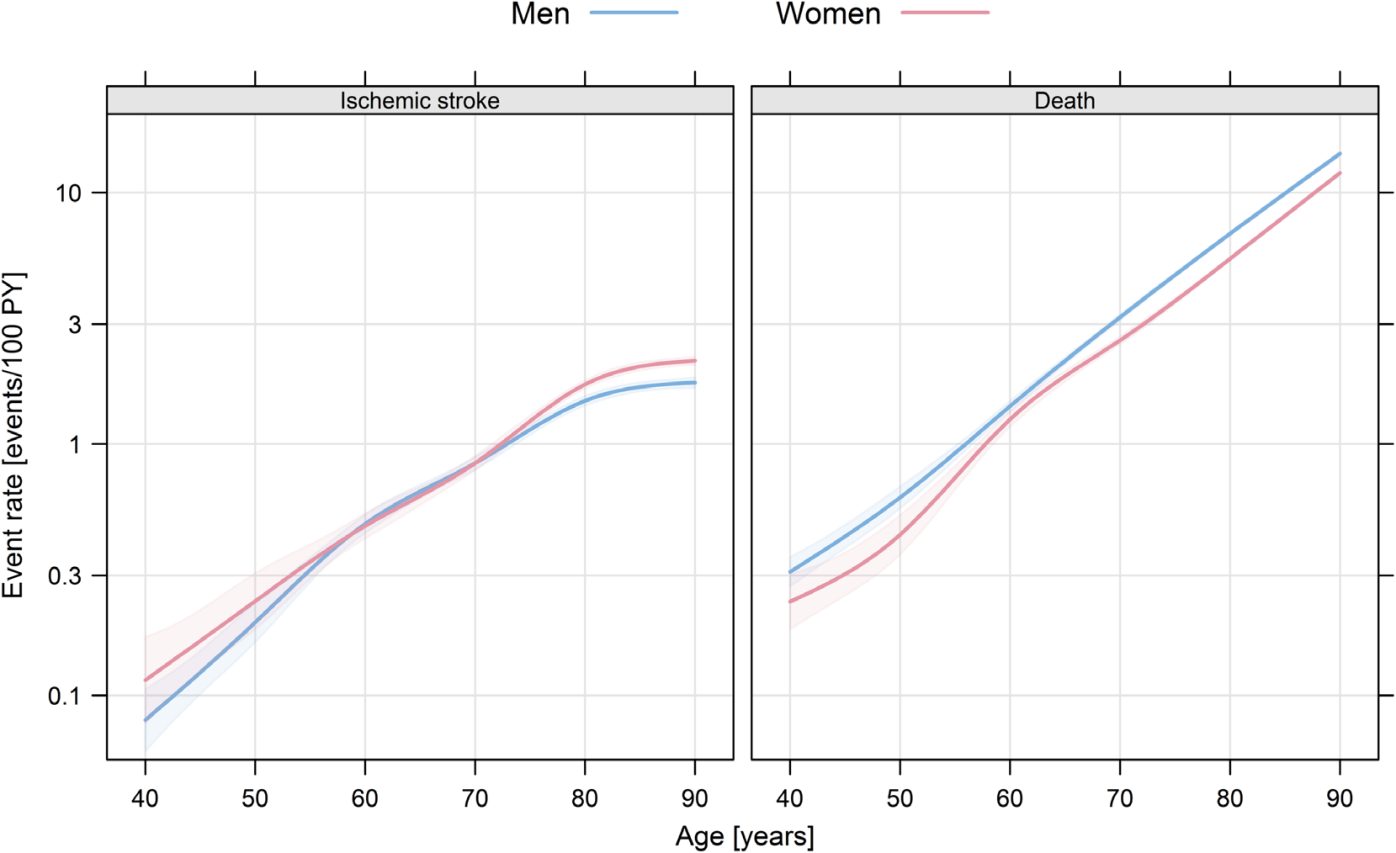
Estimated event rate of ischemic stroke (left panel) and death (right panel) in relation to age without any other CHA_2_DS_2_‐VASc risk factors. Estimated event rate of ischemic stroke (left panel) and death (right panel) in relation to age in women (red line) and men (blue line) without any other CHA_2_DS_2_‐VASc risk factors than age and sex. Estimation was done by fitting a multistate model and shaded areas represent 95% confidence intervals. PY, person‐years

Figure 2 displays the predicted event rate of ischemic stroke and death in relation to age in women and men without any other CHA_2_DS_2_‐VASc risk factor. It illustrates the minor differences in event rates of both ischemic stroke and death in women compared to men and also the higher event rates associated with higher age. In women and men at 55 to 70 years of age there were only minor differences in event rates.

### 3.1 Ischemic stroke in relation to sex and CHA_2_DS_2_‐VASc score

**Figure 3:**
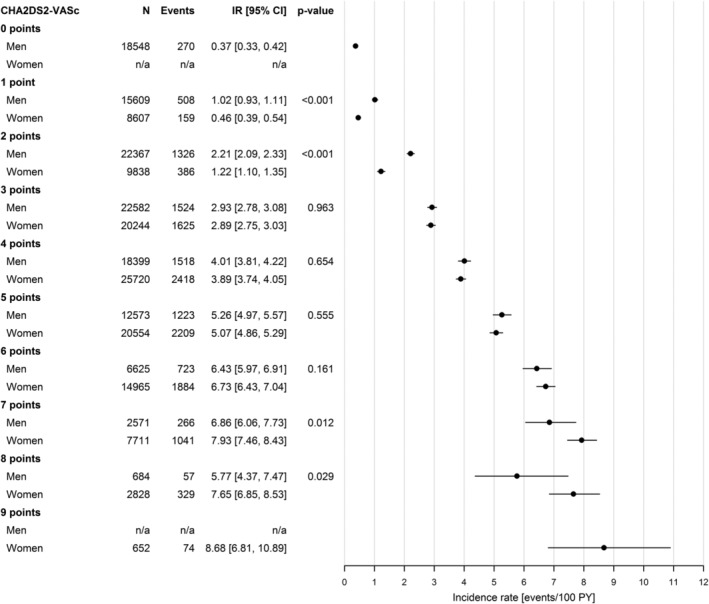
Incidence rates of ischemic stroke per 100 person‐years with 95% confidence intervals in men and women in relation to CHA_2_DS_2_‐VASc score. Incidence rates of ischemic stroke per 100 person‐years of follow‐up with 95% confidence intervals in the total group of 231 077 included patients divided by gender and CHA_2_DS_2_‐VASc score. The *P*‐value for a test of no gender difference within each CHA_2_DS_2_‐VASc score category was calculated using a Cox regression model including gender as the only covariate. CI, confidence interval; IR, incidence rate; N, number of patients; PY, person‐years

Figure 3 shows incidence rates of ischemic stroke for men and women in relation to CHA_2_DS_2_‐VASc scores. Men without CHA_2_DS_2_‐VASc risk factors had low risk for ischemic stroke, incidence rate 0.37 (CI: 0.33‐0.42). Among patients with CHA_2_DS_2_‐VASc score 1, men had higher ischemic stroke incidence than women, 1.02 (CI: 0.93‐1.11) for men and 0.46 (CI: 0.39‐0.54) for women, respectively. Similarly, for patients with CHA_2_DS_2_‐VASc score 2, men had higher ischemic stroke incidence rates than women, 2.21 (CI 2.09‐2.33) and 1.22 (1.10‐1.35), respectively. Among patients with CHA_2_DS_2_‐VASc scores 3 to 6, the differences between men and women were not statistically significant. In patients with CHA_2_DS_2_‐VASc scores 7 to 8, women instead had significantly higher risk of ischemic stroke than men.

### 3.2 Risk of ischemic stroke in relation to individual CHA_2_DS_2_‐VASc risk factors

**Figure 4:**
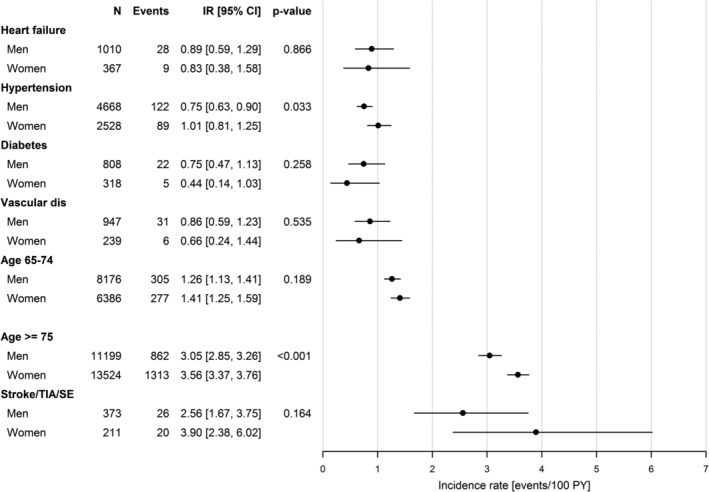
Incidence rates of ischemic stroke per 100 person‐years with 95% confidence interval in men and women with one additional non‐sex CHA_2_DS_2_‐VASc risk factor. Incidence rates of ischemic stroke per 100 person‐years of follow‐up with 95% confidence intervals in patients with one additional non‐sex CHA_2_DS_2_‐VASc risk factor divided by gender and CHA_2_DS_2_‐VASc risk factors. The *P*‐value for a test of no gender difference within each CHA_2_DS_2_‐VASc score category was calculated using a Cox regression model including gender as the only covariate. CI, confidence interval; dis, disease; IR, incidence rate; N, number of patients; PY, person‐years; SE, systemic embolism

Incidence rates of ischemic stroke in patients with only one non‐sex CHA_2_DS_2_‐VASc risk factor are shown in Figure 4. For those with only one non‐sex CHA_2_DS_2_‐VASc risk factor allotted 1 point, men had incidence rate 1.02 (CI: 0.93‐1.11) and women had 1.22 (CI: 1.10‐1.35), respectively. The incidence rates ranged from 0.44 to 1.41 in men and women with only one of either heart failure, hypertension, diabetes or vascular disease, with significant difference between men and women only in those with hypertension (Figure 4). Age 65 to 74 years was the most common non‐sex CHA_2_DS_2_‐VASc risk factor allotted 1 point, and associated with incidence rates 1.26 (CI: 1.13‐1.41) in men and 1.41 (CI: 1.25‐1.59) in women, respectively, *P* = .189. In patients with only one non‐sex CHA_2_DS_2_‐VASc risk factor allotted 2 points, the incidence rates for ischemic stroke were consistently above 2.5 events per 100 person‐years. There were significantly higher risks for ischemic stroke in women than men aged 75 years or above, but nonsignificant differences between women and men in the very small group of patients with prior stroke, transient ischemic attack, or systemic embolism (Figure 4).

## 4 DISCUSSION

In this study of patients with AF without OAC treatment, the risk of ischemic stroke and death is higher among women compared to men but this association appears to be the result of confounding by age. The difference between the sexes in risk of ischemic stroke is attenuated in the younger age groups, but the difference in ischemic stroke risk becomes more pronounced above the age of 70. The single point given for female sex in the CHA_2_DS_2_‐VASc score thereby overestimates the risk conferred to gender, while the single point given for age 65 to 74 years underestimates the risk conferred by age.

The importance and magnitude of difference in ischemic stroke risk between men and women have been vigorously debated and study results and interpretations have been inconsistent. In a previous observational Swedish cohort study, women had a moderately increased risk of ischemic stroke compared to men with an adjusted HR of 1.18 (CI: 1.12‐1.24).^5^ When divided by age, the unadjusted HR among those patients aged ≥75 years was 1.24 (CI: 1.18‐1.30), while in patients <75 years there was no significant difference in risk. Similarly, a Danish cohort study found no significant differences in stroke/thromboembolic risk in age groups <65 and 65 to 74 years, while among those aged 75 years or older, women had higher risk of stroke/thromboembolism than men (HR: 1.20, CI: 1.12‐1.28).^17^ In the GARFIELD‐AF registry, the stroke risk was elevated from the age of 70 years in women and from 65 years in men.^9^

In line with our results, a recent Danish AF cohort study found similar risks of thromboembolic events in women and men without any non‐sex CHA_2_DS_2_‐VASc risk factors, with annual absolute risks of 0.5% without OAC treatment in both women and men.^11^ This study also evaluated a CHA_2_DS_2_‐VASc risk score, thus excluding the sex category criterion, but the authors recommended against implementation of this revised risk score since it could potentially confuse prescribing physicians, unintentionally indicating that women in general do not carry excess stroke risk relative to men. An UK AF cohort study reported ischemic stroke incidence rates per 100 person‐years of 0.4 in men and 0.2 in women without any non‐sex CHA_2_DS_2_‐VASc risk factors and not on OAC treatment.^14^

In the current study, we found an overall 53% higher risk of ischemic stroke in women compared to men, but when stratified into age groups the difference in risks for women vs men was much lower in all groups. There was also an overall higher risk in the composite of stroke or death in women compared with men, but when divided by age the risk was lower in women compared to men in all age groups indicating that this difference in men and women is confounded by age. Our results are in line with the Framingham Heart Study^4^ where female sex was found to be an independent risk factor for stroke but not for a combined endpoint of stroke or death in patients with AF, which the authors assumed to be because of higher risk of mortality from other causes in men. In summary, the higher risk for ischemic stroke in women than in men seems to be lower than reflected by the CHA_2_DS_2_‐VASc score, where female sex is allotted 1 point like several clinical risk factors, irrespective of age. This is further emphasized by our results showing that the risk for ischemic stroke was similar in women and men with only one of the non‐sex CHA_2_DS_2_‐VASc risk factors heart failure, age 65 to 74 years, diabetes or vascular disease.

Previous studies have also suggested that the non‐sex risk factors of the CHA_2_DS_2_‐VASc score allotted 1 point do not carry equal risk of stroke, and that age 65 to 74 years is associated with higher risk of stroke than the other risk factors allotted 1 point.^14^, ^18^ The current European AF guidelines emphasize that OAC treatment should be considered especially in those with age 65 to 74 years as the only non‐sex CHA_2_DS_2_‐VASc risk factor. In our study, patients with age 65 to 74 years as the only non‐sex CHA_2_DS_2_‐VASc risk factor, the risk for ischemic stroke was approximately doubled compared with patients having only one of the other CHA_2_DS_2_‐VASc risk factors allotted 1 point, but still similar for women and men.

The aim of our study was to evaluate the risk for stroke and death in women and men, but not to revise the CHA_2_DS_2_‐VASc risk score, for example, without sex category as has been suggested in some recent publications.^11^, ^19^ As already pointed out, all CHA_2_DS_2_‐VASc risk factors allotted 1 point do not carry equal risk for stroke, for example, a diagnosis of hypertension or diabetes may also be associated with less increased stroke risk compared to age of 65 to 74 years,^14^, ^18^, ^20^, ^21^, ^22^ which should be taken into account in future efforts to develop new, or revise available, clinical risk scores for stroke in AF. This is further illustrated by a recent Taiwanese study where stroke risk was evaluated in relation to multiple age categories (5‐year intervals) in patients with AF and only one non‐sex CHA_2_DS_2_‐VASc risk factor.^23^ The results highlighted the continuously increased risk for stroke in relation to age, already from the age of 35 in patients with comorbidities associated with stroke risk. It also indicated that different age thresholds may apply as the tipping point for OAC treatment in patients with either of congestive heart failure, hypertension, diabetes, or vascular disease, respectively. Sex category was however not evaluated in relation to age categories.^23^

### 4.1 Study strengths and limitations

The strengths of this study comprise that it includes a very large cohort consisting of all Swedish patients with reported AF diagnosis that have been hospitalized or managed in outpatient hospital clinics nationwide with virtually no loss to follow‐up due to the unique individual civic registration numbers in Sweden. Further, the risk analysis is based on a high number of ischemic strokes and deaths, in total 17 540 ischemic strokes and 84 682 deaths. Other less well‐defined outcomes such as transient ischemic attacks or other thromboembolic events, which may be difficult to capture in registers, were not included in the analyses.

This study has limitations. It was observational and should thereby be interpreted with caution, despite careful selection of diagnosis codes for estimation of the CHA_2_DS_2_‐VASc score, the selection of robust outcomes of ischemic stroke and death, and the extensive statistical analyses. Outcomes were not adjudicated which may have led to some overestimation of event rates, although it must be considered unlikely that this would have affected the sexes differently. The inclusion of patients with AF but without OAC treatment may cause selection bias. Patients not treated with OAC are more likely to have contraindications or relative contraindications for OAC treatment due to comorbidities not accounted for in the CHA_2_DS_2_‐VASc score. Another limitation is that patients managed exclusively in primary care were not included in the cohort.

## 5 CONCLUSION

The risk of ischemic stroke was 1.5‐fold higher in women compared to men but this association appears to be the result of confounding by age. The CHA_2_DS_2_‐VASc risk score underestimates the ischemic stroke risk conferred by age 65 to 74 years, while it overestimates the risk conferred by female sex at the low risk end of the scale. These results support the current European and US AF guidelines giving equal recommendations to both men and women with only one non‐sex CHA_2_DS_2_‐VASc factor, but the importance of age of 65 to 74 years as the single risk factor for stroke should be emphasized.

## CONFLICT OF INTEREST

The authors declare no potential conflict of interests.

## Supporting information


**Figure S1.** Flowchart of inclusion/exclusion process of patients.
**Table S1.** List of ICD‐10 codes and ATC medication codes used to estimate CHA_2_DS_2_‐VASc risk factors of included patients and to exclude patients with mitral stenosis or a prosthetic heart valve and patients with oral anticoagulation treatment.
**Table S2.** Baseline characteristics of all included patients by gender, with percentage and number of patients in each age group and CHA_2_DS_2_‐VASc risk factor group.
