# Supplementary material for: Risk of ischemic stroke and utility of CHA2DS2‐VASc score in women and men with atrial fibrillation
Source: Clin Cardiol. 2019 Sep 6;42(10):1003–9. doi: 10.1002/clc.23257 (PMC6788468; doi:10.1002/clc.23257)

**Online-Only Supplemental material**

**Table 1. List of ICD-10 codes and ATC medication codes used to estimate CHA_2_DS_2_-VASc risk factors of included patients and to exclude patients with mitral stenosis or a prosthetic heart valve and patients with oral anticoagulation treatment.**

|  | **ICD-10 codes** | **ATC codes** |
| --- | --- | --- |
| CHA2DS2-VASc risk factor |  |  |
| Heart failure | I11.0, I13.0, I13.2, I42, I43, I50, K76.1 | - |
| Hypertension | I10-I13, I15 | - |
| Diabetes | E10-E14 | A10A, A10B, A10X |
| Stroke, TIA, systemic embolism | G45, I63-I64, I74 | - |
| Vascular disease | I21, I22, I25.2, I70-I73 | - |
| Exclusion of patients with mitral stenosis or prosthetic heart valve | I05.0, I05.2, I34.2, Z95.2, Q23.2 | - |
| Exclusion of patients with treatment with oral anticoagulation | - | B01AA03, B01AA04, B01AE07, B01AF01, B01AF02 |

ICD-10=International Classification of Diseases - 10^th^ Revision, ATC=Anatomical Therapeutic Chemical Classification System, TIA=transient ischemic attack.

**Table 2. Baseline characteristics of all included patients by gender, with percentage and number of patients in each age group and CHA_2_DS_2_-VASc risk factor group.**

|  | **All patients** | **Men** | **Women** |
| --- | --- | --- | --- |
|  | N = 231 077 | N = 119 958 | N = 111 119 |
| Age 18 - 64 years | 20.0% (46 260) | 26.7% (31 987) | 12.8% (14 273) |
| Age 65 - 74 years | 19.6% (45 260) | 22.9% (27 411) | 16.1% (17 891) |
| Age ≥75 years | 60.4% (139 515) | 50.5% (60 560) | 71.1% (78 955) |
| Heart failure | 28.5% (65 905) | 26.7% (32 011) | 30.5% (33 894) |
| Hypertension | 48.4% (111 744) | 44.3% (53 149) | 52.7% (58 595) |
| Diabetes | 17.2% (39 648) | 18.0% (21 583) | 16.3% (18 065) |
| Stroke/TIA/SE | 18.7% (43 141) | 17.0% (20 379) | 20.5% (22 762) |
| Vascular disease | 24.1% (55 663) | 26.5% (31 737) | 21.5% (23 926) |

N=number of patients, TIA=transient ischemic attack, SE=systemic embolism.

**Figure 1. Flowchart of inclusion/exclusion process of patients.**

Flowchart of inclusion/exclusion process of patients. AF=atrial fibrillation, N=number of patients, OAC=oral anticoagulation treatment.


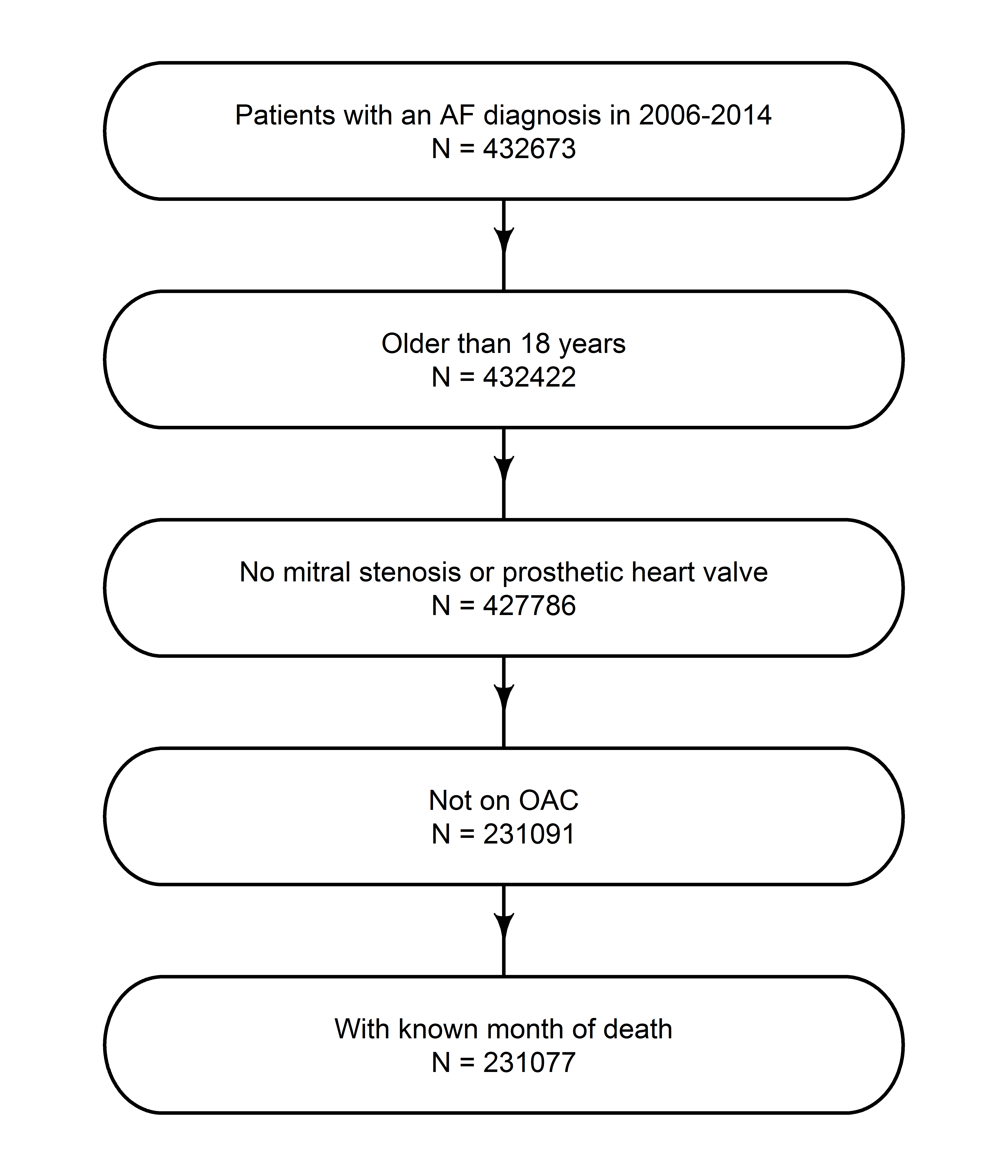

Supplement: Supplementary file 1 — Figure S1. Flowchart of inclusion/exclusion process of patients. Table S1. List of ICD‐10 codes and ATC medication codes used to estimate CHA2DS2‐VASc risk factors of included patients and to exclude patients with mitral stenosis or a prosthetic heart valve and patients with oral anticoagulation treatment. Table S2. Baseline characteristics of all included patients by gender, with percentage and number of patients in each age group and CHA2DS2‐VASc risk factor group. [file CLC-42-1003-s001.docx]
